# Survey of the differences between Migraine and MOH pre-existing migraine

**DOI:** 10.1186/1129-2377-14-S1-P176

**Published:** 2013-02-21

**Authors:** H Igarashi

**Affiliations:** 1 Department of Internal Medicine Yokohama Dental and Medical Clinic Kanagawa Dental College, Japan

## Objective

Most patients with medication-overuse headache (MOH) have a history of migraine. The author evaluated patients with migraine and patients with MOH pre-existing migraine.

## Subjects and methods

 All patients completed questionnaires and were interviewed by the author. The Headache Impact Test (HIT6), Body Mass Index (BMI), and Zung Self-rating Depression Scale (SDS) were investigated. Headache diagnosis was based on ICHD-21) and Appendix 8.2 Medication overuse headache[2]).

## Results

During 7 years, 3754 patients (901 men and 2853 women, 5-93 years old) visited the clinic. At the first visit, 2034 patients (425 men and 1609 women) were diagnosed with migraine, 295 (43 men and 252 women) with migraine and frequent tension-type headache, 98 (18 men and 80 women) with migraine and chronic tension-type headache and 560 (103 men and 457 women) with MOH. Of 560 patients with MOH, MOH pre-existed migraine in 447 patients (67 men and 380 women). The average HIT6 was 63.1±6.7 for the migraine group and 65.8 for MOH pre-existing migraine. The average BMI was 20.8 for the migraine group and 21.3 for MOH pre-existing migraine. The average SDS was 40.9 for the migraine group and 44.8 for MOH pre-existing migraine. Patients with MOH pre-existing migraine experienced more occipital pain than patients with migraine (66% vs 52%). There were no differences in associated symptoms such as nausea, vomiting, photo/phonophobia between the two groups. Patients with MOH pre-existing migraine had more neck/shoulder stiffness and/or pain and reported more trigger factors such as neck/shoulder stiffness or pain, crowded places, weather changes, lack of sleep or oversleeping psychological stress than the migraine group. Patients with MOH pre-existing migraine had greater psychological disorder than the migraine group (13% vs 6%).

## Conclusions

Neck/shoulder stiffness and/or pain is reported more often in patient with MOH pre-existing migraine.
